# Identification of Temporal and Region-Specific Myocardial Gene Expression Patterns in Response to Infarction in Swine

**DOI:** 10.1371/journal.pone.0054785

**Published:** 2013-01-25

**Authors:** Cristina Prat-Vidal, Carolina Gálvez-Montón, Lara Nonell, Eulàlia Puigdecanet, Laura Astier, Francesc Solé, Antoni Bayes-Genis

**Affiliations:** 1 Imperial College Research Ethics Committee (Heart Failure and Cardiac Regeneration) Research Program, Health Sciences Research Institute Germans Trias i Pujol. Cardiology Service, Hospital Universitari Germans Trias i Pujol, Badalona (Barcelona), Spain; 2 Servei d'Anàlisi de Microarrays, Institut Hospital del Mar d'Investigacions Mèdiques, Barcelona, Spain; 3 Laboratori de Citogenètica Molecular, Servei de Patologia, Hospital del Mar, Barcelona, Spain; 4 Department of Medicine, University Autonomous of Barcelona, Barcelona, Spain; University Hospital Freiburg, Germany

## Abstract

Molecular mechanisms associated with pathophysiological changes in ventricular remodelling due to myocardial infarction (MI) remain poorly understood. We analyzed changes in gene expression by microarray technology in porcine myocardial tissue at 1, 4, and 6 weeks post-MI.

MI was induced by coronary artery ligation in 9 female pigs (30–40 kg). Animals were randomly sacrificed at 1, 4, or 6 weeks post-MI (n = 3 per group) and 3 healthy animals were also included as control group. Total RNA from myocardial samples was hybridized to GeneChip® Porcine Genome Arrays. Functional analysis was obtained with the Ingenuity Pathway Analysis (IPA) online tool. Validation of microarray data was performed by quantitative real-time PCR (qRT-PCR).

More than 8,000 different probe sets showed altered expression in the remodelling myocardium at 1, 4, or 6 weeks post-MI. Ninety-seven percent of altered transcripts were detected in the infarct core and 255 probe sets were differentially expressed in the remote myocardium. Functional analysis revealed 28 genes de-regulated in the remote myocardial region in at least one of the three temporal analyzed stages, including genes associated with heart failure (HF), systemic sclerosis and coronary artery disease. In the infarct core tissue, eight major time-dependent gene expression patterns were recognized among 4,221 probe sets commonly altered over time. Altered gene expression of ACVR2B, BID, BMP2, BMPR1A, LMNA, NFKBIA, SMAD1, TGFB3, TNFRSF1A, and TP53 were further validated.

The clustering of similar expression patterns for gene products with related function revealed molecular footprints, some of them described for the first time, which elucidate changes in biological processes at different stages after MI.

## Introduction

Myocardial infarction (MI), generally due to coronary artery occlusion, results in an inappropriate oxygen supply to the downstream myocardium [1]. A massive loss of cardiac muscle occurs and the left ventricle, in an attempt to maintain normal pump function, undergoes structural and functional adaptations that have been termed as left ventricular (LV) remodelling, ultimately leading to heart failure (HF) [2]–[4].

The molecular mechanisms underlying LV remodelling and its progression toward HF remain poorly understood. Several studies have analyzed the molecular pathways of LV remodelling, albeit most of them has been performed in small animal models [5]–[7] or has only provided data of individual genes and proteins [8]. Moreover, significant differences exist with regard to cardiac characteristics such as heart rate, oxygen consumption, adrenergic receptor ratios, and response to loss of regulatory proteins, when mice are contrasted to humans [9]. Therefore, large animal models which more closely approximate human physiology, function, and anatomy, are essential to develop the discoveries from murine models into clinical therapies and interventions for HF. The coronary artery pattern and distribution of blood supply in swine is remarkably similar to that of humans, more so than any other mammal [10]–[12]. Thus, the porcine model of MI is extensively used to better understand functional, structural, and molecular changes associated with clinical ischemic heart disease.

The availability of microarray technology has led to the simultaneous analysis of thousands of genes in a given tissue and may identify genes responsible for the relevant phenotype [13]. In the past decade, the development of pig cDNA microarray sequences generated a tremendous increase in porcine transcriptomic data. Therefore, in order to investigate molecular alterations caused by MI we studied changes in gene expression by microarray analysis of infarcted and non-infarcted (remote myocardium) porcine myocardial samples at different stages after MI (1, 4, and 6 weeks).

## Materials and Methods

This study was approved by the Animal Experimentation Unit Ethical Committee of the Catalan Institute of Cardiovascular Sciences (ICCC) (Permit Number: 4563; Departament de Medi ambient i Habitatge (DMAH), Generalitat de Catalunya) and complies with all guidelines concerning the use of animals in research and teaching as defined by the *Guide For the Care and Use of Laboratory Animals* (NIH Publication No. 80-23, revised 1996).

### Myocardial infarction model

For this study, 9 female crossbreed Landrace×Large White pigs (30–40 kg) were premedicated with azaperone (10 mg/kg, intramuscularly (IM)) followed by pentobarbital sodium (15 mg/kg, intravenously (IV)). Animals underwent endotracheal intubation, and anaesthesia was maintained by 2% isoflurane inhalation. During intervention, fentanyl (0.75 mg/kg/45 min, IV) was used as analgesic and 1.5 mg/kg atracurium besylate intravenous bolus was administered inducing muscular relaxation. After left lateral thoracotomy through the fourth intercostal space, MI was induced by a permanent double-ligation of the first marginal branch of the circumflex artery, as previously described [14]. Tulatromicin (2.5 mg/kg, IM) was administered at the end of the intervention as antibiotic prophylaxis and a transdermal fentanyl patch was applied to allow analgesic post-operative care. Surgical procedures were monitored with ECG registration and capnography, pulse oximetry, non-invasive arterial blood pressure, and temperature measurements.

Animals were randomly sacrificed with an intravenous overdose infusion of potassium chloride solution at 1 week (n = 3), 4 weeks (n = 3), or 6 weeks (n = 3) after MI. Three paired myocardial samples from infarct core and non-infarcted remote myocardium were analyzed at each temporal stage. Myocardial samples from healthy animals (n = 3) were included as physiological condition.

### Tissue collection and RNA extraction

After sternotomy, hearts were immediately excised and washed in ice-cold buffered saline solution to remove any residual blood. Biopsies from infarct core (middle of the scar of the left ventricle), remote myocardium (non-infarcted interventricular septum), and control myocardium (healthy animals) were selected and immediately collected in Allprotect Tissue Reagent (Qiagen) at room temperature to ensure RNA stabilization of harvested tissue samples.

RNeasy Fibrous Tissue Mini Kit (Qiagen) was used to isolate total RNA from tissue. RNA purity and integrity, assessed both by spectrophotometry (NanoDrop ND-1000, NanoDrop Technologies) and nanoelectrophoresis (2100 Bioanalyzer, Agilent Technologies), were optimal for microarray experiments (RNA purity, A260/280>2.0 and A260/230>1.4; RNA integrity number 6.9–9.3).

### Microarray gene expression analysis

Microarray expression profiles were obtained using the GeneChip® Porcine Genome Array (Affymetrix). Briefly, 200 ng of total RNA from each sample was processed, labeled, fragmented, and hybridized to GeneChip® according to the Affymetrix GeneChip® 3′ IVT Express Kit User Manual. Washed and stained arrays were scanned using an Affymetrix GeneChip® Scanner 3000 7 G, generating. CEL files for each array. Raw expression values obtained from. CEL files for each array were pre-processed using the robust multi-chip average (RMA)-normalization method [15] obtaining a total of 24,123 probe sets on a log_2_ basis. Data were subjected to a first non-specific filtering by selecting probe sets with normalized intensity above 3 in at least 10 samples to remove those transcripts that show little or no variability. Selection of differentially expressed transcripts was based on a linear model for microarray analysis (LIMMA) with empirical Bayes modification for the variance estimates, as described previously [16]. Correction for multiple comparisons was performed using FDR [17] and only probe sets with adjusted *P* value less than 0.05 were selected for subsequent analysis. A total of 11,290 genes were annotated by blasting the microarray sequences to the human genome, as previously described [18]. Data analysis was performed in R (version 2.11.1) with standard Bioconductor packages. A total of six lists of differentially expressed probe sets were generated by comparing microarray expression profiles of remote myocardium or infarct core region with respect microarray expression profile of control myocardium at 1, 4, and 6 weeks post-MI. Results are presented in terms of log_2_ fold change (FC) with 95% confidence interval (CI), *P* value and adjusted *P* value, as obtained by LIMMA package.

For functional analysis, the six lists of differentially regulated probe sets were uploaded into the Ingenuity Pathway Analysis (IPA) online tool (Ingenuity System Inc, www.ingenuity.com). The ‘Core Analysis’ function included in IPA was used to interpret data in the context of biological processes, pathways, and networks. Eight different temporal expression patterns identified in the analysis were derived by combining the sign of the log_2_ FC variable from paired comparisons between infarct core versus remote myocardium at three temporal analyzed stages. Microarray data have been deposited in the Gene Expression Omnibus (GEO, http://www.ncbi.nlm.nih.gov/geo/) database with series accession number GSE34569.

### Quantitative real-time PCR

To confirm microarray data, ten of the de-regulated genes in the infarct core tissue with porcine cDNA sequences commercially available, were selected for further validation by quantitative real-time PCR (qRT-PCR). Random hexamers and the Script™ One-Step RT-PCR Kit (Bio-Rad) were used to synthesize first strand cDNA for each sample from 2 µg of RNA. 2 µl of cDNA were amplified in a final volume of 50 µl containing 25 µl TaqMan® 2× Universal PCR Master Mix and 2 µl of each porcine FAM-labelled TaqMan® Gene Expression Assay (Applied Biosystems). The used primers are listed in Table S1. Data were collected and analyzed on the ABI Prism® 7000 Sequence Detection System (Applied Biosystems). Relative quantification was determined by normalizing the expression for each gene to PGK1 gene following the 2^−ΔΔCt^ method [19]. PGK1 was selected as a housekeeping gene among three candidate genes (PGK1, GUSB, and GAPDH), since it showed the most constant expression levels for both physiological and pathological samples. All measurements were performed in duplicate in ten separate runs and expressed as mean ± sd. *P* values for significance were analyzed using Student's t-test. Spearman's correlation was computed to assess concordance between qRT-PCR and microarray data.

## Results

### MI alters gene expression in the whole heart

**Figure 1:**
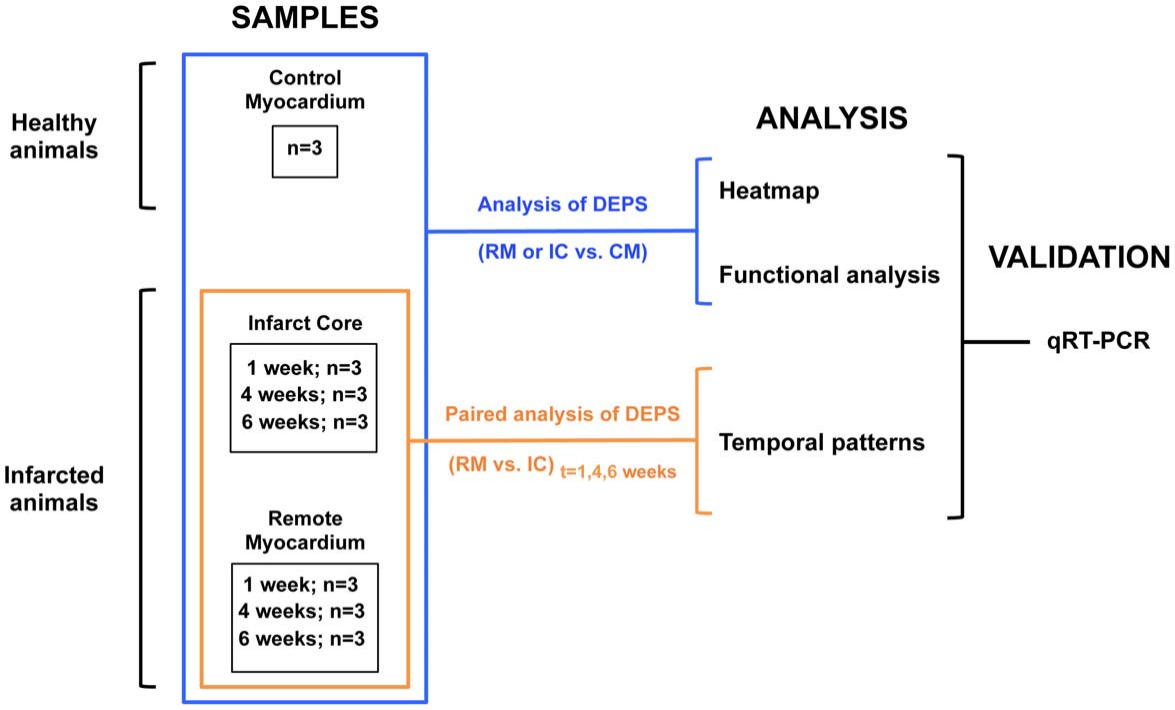
Study design. DEPS = differentially expressed probe sets; CM = control myocardium; IC = infarct core; RM = remote myocardium; qRT-PCR = quantitative real-time PCR.

In order to analyze changes in gene expression caused by infarct injury in the whole heart, microarray expression profiles of non-infarcted remote myocardium or infarct core samples at each temporal post-MI stage (1, 4, and 6 weeks) were compared with microarray expression profile of control myocardial samples from healthy animals (physiological condition) (Fig. 1). A total of six lists of differentially expressed probe sets were obtained for all comparisons (Tables S2, S3, S4, S5, S6, S7).

**Figure 2:**
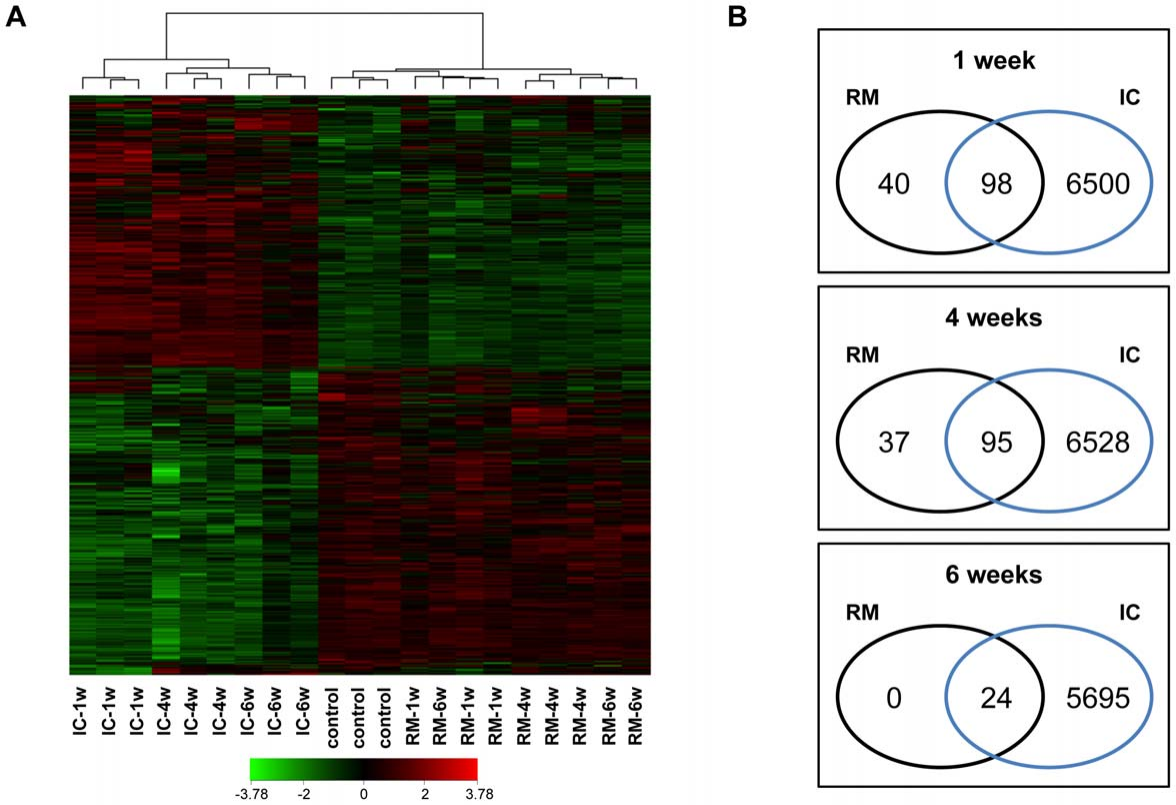
Differentially clustered gene expression patterns in MI. **a** Probe sets for genes significantly regulated (adjusted *P* value<0.05 by significance analysis of microarrays) at 1, 4, or 6 weeks post-MI were selected, clustered, and reported in a heat map. Each row represents a different probe set, and columns pertain to data collected at three temporal stages after surgery. Control myocardium from healthy animals was also included. Normalized data values, displayed in red and green shades, are represented according to the colour scale shown (bottom). **b** Venn diagrams summarizing similarities and differences between tissue regions at each temporal stage, using significant probe sets in **a**. IC = infarct core; RM = remote myocardium; w = weeks; n = 3 per group.

A compressed view of the cluster analysis of all expressed probe sets showing relative differential expression in infarct core and remote myocardium at 1, 4, and 6 weeks is shown by the heat map (Fig. 2A). In this hierarchical clustering algorithm, all the 9 infarct core and all the 9 remote myocardial samples formed two distinct clusters, separately of the control samples cluster, corresponding to physiological condition (Fig. 2A). In addition, except one remote myocardial specimen from 6 weeks group, the samples also clustered by temporal post-MI stage.

A total of 8,619 differentially expressed probe sets, which correspond to 6,108 unique annotated HGNC genes, were detected in the infarcted porcine heart (Fig. 2A). Fig. 2B shows the number of transcripts differentially expressed by the infarct core and remote myocardium at each temporal analyzed stage. Venn's diagrams show overlapped regulated probe sets between both tissue regions diminishing over time (98, 95, and 24 at 1 week, 4 weeks, and 6 weeks, respectively) (Fig. 2B). Among 223 overlapped transcripts (Fig. S1A), IPA analysis recognized 193 genes, some of them related to cardiac arteriopathy (AMY2B, DOCK9, FAM107B, FAM78B, HBA2, LRRC17, NPR1, and PRKCH), cardiac arrhythmia (MAOA and PP1CA), and HF (NPR1). Remarkably, only 2 genes (PAQR3 and FRK) were differentially expressed in the infarcted heart at all temporal stages (Fig. S1B).

### Altered genes in the remote myocardium due to an infarct event

MI caused altered expression of 255 probe sets in the remote myocardial tissue at 1, 4, or 6 weeks post-MI (Fig. S1A). Using IPA software, 28 unique genes differentially expressed due to infarct damage were identified exclusively in the remote myocardium in at least one of the three temporal analyzed stages (Table 1), and only 1 gene (CCDC15) was permanently de-regulated over time in this region. A temporal stage analysis revealed early at 1 week post-MI, 8 cardiovascular-related genes (ADAMTS3, EDNRB, ERG, ILK, RASSF1, RRAD, SRF, and ZSWIM6) among 40 altered transcripts in the remote myocardial region (Fig. 2B and Table 2). At 4 weeks post-MI, 4 genes (DOCK9, MBNL1, RRAD, and ZSWIM6) by all of 37 unique altered transcripts were related to cardiovascular disease. Lastly, at 6 weeks post-MI no unique genes altered only in the remote myocardium were found, although IPA identified 3 genes (MAP2K5, MBNL1, and NFIA) of 24 shared probe sets by remote myocardium and infarct core regions associated with cardiac arteriopathy (Fig. 2B and Table 2).

**Table 1:** Unique genes differentially regulated in the remote myocardium at 1, 4, or 6 weeks post-MI. FC = fold change; w = weeks.

		1 week		4 weeks		6 weeks	
Gene Symbol	Gene Name	Log_2_FC	adj *P* value	Log_2_FC	adj *P* value	Log_2_FC	adj *P* value
ATXN7	ataxin 7	−0.32	n.s.	−0.80	3.5E-02	−0.71	n.s.
CCDC73	coiled-coil domain containing 73	−0.87	3.9E-02	−0.22	n.s.	−0.54	n.s.
CNTF	ciliary neurotrophic factor	−0.03	n.s.	1.41	3.3E-02	0.72	n.s.
DLC1	deleted in liver cancer 1	0.97	1.7E-02	0.75	3.3E-02	0.84	n.s.
DYNLT1	dynein, light chain, Tctex-type 1	0.72	n.s.	1.03	1.5E-02	0.32	n.s.
EDNRB	endothelin receptor type B	−1.29	4.3E-02	−0.74	n.s.	−0.70	n.s.
ENPEP	glutamyl aminopeptidase (aminopeptidase A)	−1.39	1.6E-02	−0.58	n.s.	−0.80	n.s.
ERG	v-ets erythroblastosis virus E26 oncogene homolog (avian)	−1.05	4.7E-02	−0.63	n.s.	−0.87	n.s.
GADL1	glutamate decarboxylase-like 1	1.03	3E-02	0.31	n.s.	0.55	n.s.
HECA	headcase homolog (Drosophila)	−0.58	1.7E-02	−0.28	n.s.	−0.26	n.s.
HSPA14	heat shock 70 kDa protein 14	0.66	4.7E-02	0.13	n.s.	0.32	n.s.
IQCK	IQ motif containing K	−1.18	3.1E-02	−0.52	n.s.	−0.71	n.s.
MEI1	meiosis inhibitor 1	0.43	n.s.	1.12	4.7E-02	0.39	n.s.
MGAT4B	mannosyl (alpha-1,3-)-glycoprotein beta-1,4-N-acetylglucosaminyltransferase, isozyme B	0.65	4.7E-02	0.34	n.s.	0.10	n.s.
NASP	nuclear autoantigenic sperm protein (histone-binding)	1.15	4.7E-02	0.94	n.s.	0.45	n.s.
NR4A2	nuclear receptor subfamily 4, group A, member 2	1.01	n.s.	1.85	2.5E-02	−0.41	n.s.
PELI1	pellino homolog 1 (Drosophila)	−1.02	1.4E-02	−0.97	1.5E-02	−0.85	n.s.
PIK3C2A	phosphoinositide-3-kinase, class 2, alpha polypeptide	−0.90	1E-02	−0.84	4.1E-02	−0.44	n.s.
PKIG	protein kinase (cAMP-dependent, catalytic) inhibitor gamma	0.71	3.4E-02	0.75	2.4E-02	0.45	n.s.
PLIN2	perilipin 2	−1.87	n.s.	0.18	n.s.	−0.11	n.s.
PTPRG	protein tyrosine phosphatase, receptor type, G	−0.64	4.1E-02	−0.03	n.s.	−0.40	n.s.
RASSF1	Ras association (RalGDS/AF-6) domain family member 1	−0.84	3E-02	−0.66	n.s.	−0.53	n.s.
RRAD	Ras-related associated with diabetes	1.12	4E-02	1.76	4.7E-03	1.02	n.s.
SENP6	SUMO1/sentrin specific peptidase 6	−0.08	n.s.	−0.73	2.4E-02	−0.41	n.s.
SETD7	SET domain containing (lysine methyltransferase) 7	−1.10	1.1E-02	−0.70	n.s.	−0.53	n.s.
SLC29A1	solute carrier family 29 (nucleoside transporters), member 1	0.22	n.s.	0.94	1.9E-02	0.27	n.s.
SMTNL2	smoothelin-like 2	1.32	3.8E-02	1.13	n.s.	0.76	n.s.
ZSWIM6	zinc finger, SWIM-type containing 6	−0.81	2.4E-02	−0.86	1.6E-02	−0.64	n.s

**Table 2:** Cardiovascular-related genes altered in the remote myocardium at 1, 4, or 6 weeks post-MI. w = weeks.

Gene Symbol	Gene Name	Temporal Stage	Function Annotation
ADAMTS3	ADAM metallopeptidase with thrombospondin type 1 motif, 3	1 w	coronary artery disease
DOCK9	dedicator of cytokinesis 9	4 w	coronary artery disease
EDNRB	endothelin receptor type B	1 w	heart failure, congestive heart failure, congenital heart disease, systemic esclerosis, left ventricular dysfunction, secondary pulmonary hypertension, pulmonary hypertensive arterial disease
ERG	v-ets erythroblastosis virus E26 oncogene homolog (avian)	1 w	systemic esclerosis
ILK	integrin-linked kinase	1 w	congenital outflow tract obstruction
MAP2K5	mitogen-activated protein kinase kinase 5	6 w	coronary artery disease
MBNL1	muscleblind-like (Drosophila)	4 w, 6 w	coronary artery disease
NFIA	nuclear factor I/A	6 w	coronary artery disease
RASSF1	Ras association (RalGDS/AF-6) domain family member 1	1 w	heart failure
RRAD	Ras-related associated with diabetes	1 w, 4 w	heart failure
SRF	serum response factor (c-fos serum response element-binding transcription factor)	1 w	cardiac hypertrophy
ZSWIM6	zinc finger, SWIM-type containing 6	1 w, 4 w	coronary artery disease

Interestingly, a multiple comparison between differentially expressed genes in the remote myocardial region at 1, 4, and 6 weeks after MI, highlighted common function categories associated with cardiac disease (cardiac arteriopathy, HF, and cardiac necrosis and cell death) sharing some genes altered over time (Table 3). Finally, IPA upstream regulator analysis did not reveal any transcription regulator (TR) altered in the remote myocardium at 1, 4, or 6 weeks post-MI.

**Table 3:** Functional categories related to cardiac disease commonly altered over time in the remote myocardium. w = weeks.

Category	Function Annotation	Temporal Stage	Related Genes	*P* value	Upregulated Genes	Downregulated Genes
Cardiac arteriopathy	coronary artery disease	1 w	17	4.4E-03	ADAMTS3, LRRC17, NEBL	AHCYL2, CREB5, DDR2, DOCK9, FAM107B, FAM78B, FMO1, FSTL4, KIAA1462, NCOA7, NHSL1, NPR1, PRKCH, ZSWIM6
	coronary artery disease	4 w	14	6.3E-02	AMY2B, MAP4, NAV2, PSMF1, SLC39A11, SPTBN1	COL11A1, DDR2, DOCK9, MBNL1, PPP1R12B, SPAG16, STK39, ZSWIM6
	coronary artery disease	6 w	3	1.7E-01	-	NFIA, MBNL1, MAP2K5
Heart failure	heart failure	1 w	3	1.2E-01	RRAD	RASSF1, NPR1
	heart failure	4 w	2	3.6E-01	RRAD, AMY2B	-
	cardiac decompensation of heart	6 w	1	2.1E-03	-	DUSP6
Cardiac necrosis/cell death	cell death of cardiomyocytes	1 w	1	4E-01	ZYX	-
	cell death of cardiomyocytes	4 w	2	9.9E-02	THBS2, MAOA	-
	apoptosis of heart	6 w	1	5.4E-03	-	MAP2K5

### Altered genes in the infarct core region after MI

**Figure 3:**
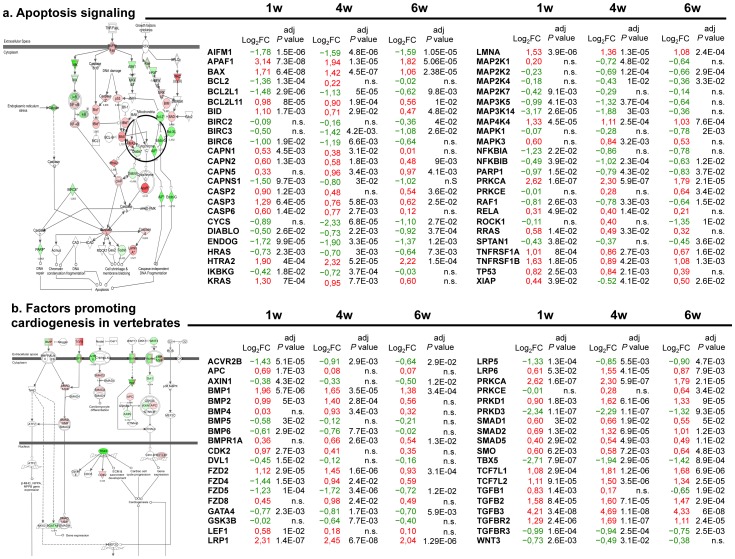
Altered canonical pathways in the infarct core tissue commonly identified at 1, 4, and 6 weeks post-MI (1). IPA software recognized differentially expressed genes from five cardiovascular-related canonical pathways at the three temporal analyzed stages. Schematically representation at 1 week post-MI is detailed for **a** apoptosis signalling and **b** factors promoting cardiogenesis canonical pathways (left panels), showing both upregulated and downregulated molecules (red or green filled, respectively). Genes with their corresponding log_2_ FC and adjusted *P* value at 1, 4, and 6 weeks post-MI are also listed. w = weeks; red value = upregulated gene; green value = downregulated gene.

In the infarct core tissue, we detected 8,587 probe sets, corresponding to 6,085 unique annotated HGNC genes, differentially expressed at 1, 4, or 6 weeks post-MI (Fig. S1A). Several extracellular matrix (ECM) and adhesion molecules genes (e.g. collagens, integrins, matrix metalloproteinases, and metalloproteinases inhibitors) were found upregulated in all temporal analyzed stages and lipid metabolism genes were found repressed over time (Tables S2, S3, S4, S5, S6, S7). IPA identified 277, 270, and 238 altered canonical pathways after 1, 4, and 6 weeks, respectively, with 219 of these pathways commonly altered over time. The five main cardiovascular-related canonical pathways commonly found in this tissue region at the temporal post-MI examined stages were: apoptosis signalling, factors promoting cardiogenesis in vertebrates, hypoxia signalling in the cardiovascular system, human embryonic stem cell pluripotency, and cardiac hypertrophy signalling (Fig. 3A–B and Fig. S2). Fig. 3 lists differentially expressed genes from apoptosis signalling and factors promoting cardiogenesis canonical pathways with their corresponding log_2_ FC and adjusted *P* values. In the apoptosis pathway, changes in gene expression affected 48 genes, including 22 overexpressed pro-apoptotic and 22 downregulated anti-apoptotic genes at all time points. The remaining four genes (BCL2, MAP2K1, PRKCE, and ROCK1) were both up- and downregulated in a time-dependent manner, according to infarct progression (Fig. 3A). Among factors promoting cardiogenesis, 23 of the 38 altered genes were upregulated and 13 downregulated; the 2 remaining genes (FZD4 and PRKCE) decreased at 1 week and increased at both 4 and 6 weeks (Fig. 3B).

A further analysis in the infarct core region revealed differentially expressed TRs with altered genes downstream at 1,4, and 6 weeks post-MI (20, 23, and 16 altered TRs, respectively) (Table S8). Interestingly, among these altered TRs, 13 of them were shared in all temporal analyzed stages: JARID1B, MBD2, NOTCH3, SMAD1, and TWIST1 were permanently upregulated although EPAS1, HEY1, HEY2, JUN, MYOCD, PPARD, PPARGC1A, and SATB1 were persistently downregulated over time.

### Temporal gene expression patterns identified in the infarct core tissue post-MI

**Figure 4:**
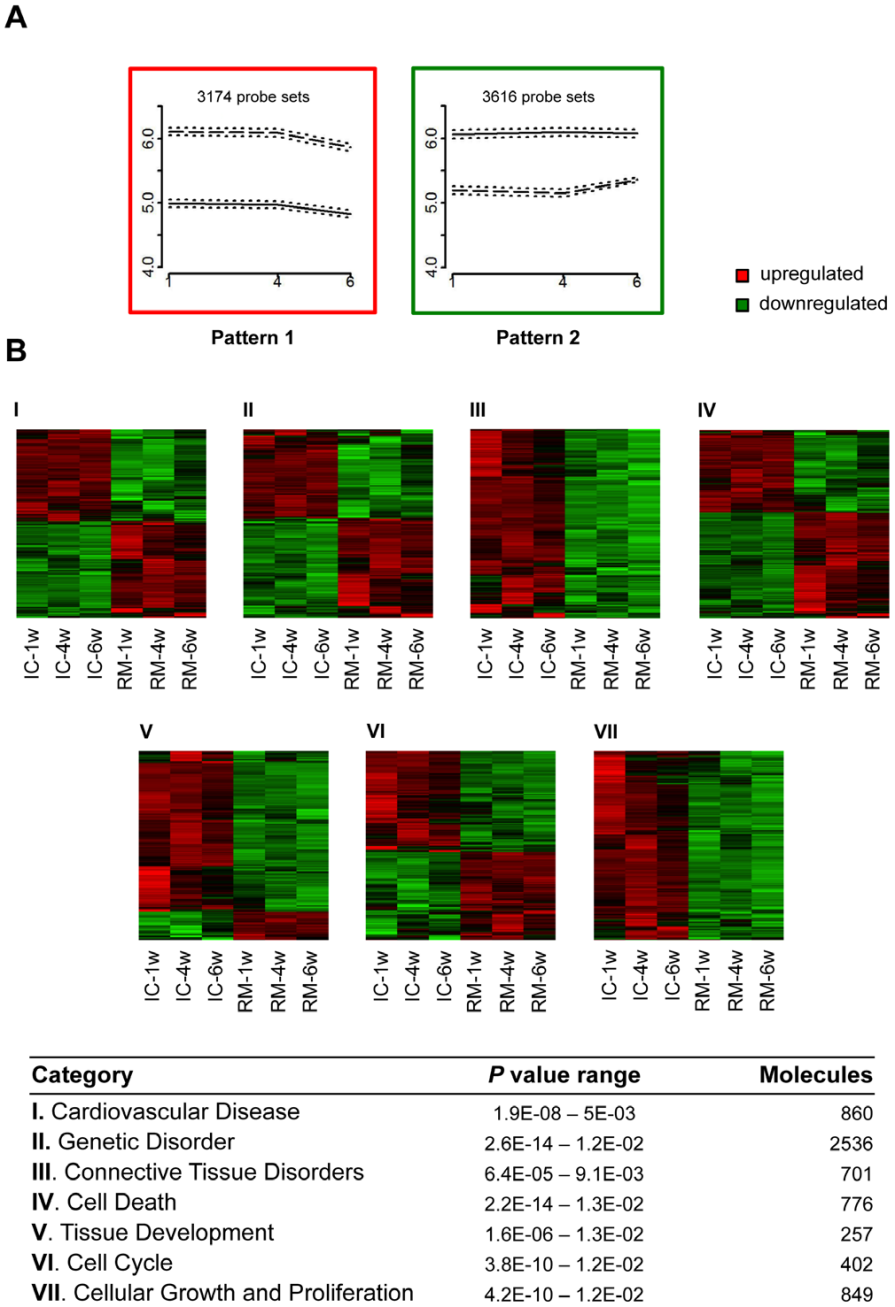
Top temporal patterns, comprising the majority of differentially expressed genes in infarct core tissue, clustered within function categories. **a** Probe sets with a similar sustained expression over time in infarct core are shown depending on the regular upregulation (pattern 1, graph framed in red) or downregulation (pattern 2, graph framed in green). In both graphs, mean of log_2_ FC for probe sets with 95% CI were plotted. Solid line = remote myocardium, dashed line = infarct core and dotted lines = 95% CI for mean. X axis relates to weeks and Y axis relates to log_2_ FC. **b** Heat maps of functional clusters from patterns, comparing remote myocardium and infarct core gene expressions. Each row represents a different probe set, and columns pertain to remote myocardium or infarct core samples at temporal stages specified. *P* value range and number of molecules pertaining to each function category identified by IPA are detailed. IC = infarct core; RM = remote myocardium; w = weeks.

A total of 8,619 differentially expressed probe sets were detected in the infarcted porcine heart (Fig. 2A). Among these differentially expressed transcripts eight main time-dependent gene patterns were identified on a paired-base method for each temporal stage (Fig. 1). Patterns 1 and 2 identified sustained gene up- or downregulation over time, respectively; 3,174 probe sets were upregulated and 3,616 transcripts were downregulated at the studied stages of infarct progression (Fig. 4A). A subsequent functional analysis of patterns 1 and 2 transcripts identified genes related to several functional categories, including cardiovascular disease (913 genes), genetic and connective tissue disorders (3,119 genes), cell death (776 genes), tissue development (203 genes), cell cycle (414 genes), and cellular growth and proliferation (528 genes). Fig. 4B shows heat maps of functional clusters for each category. Remarkably, all genes related to ischemia and atherosclerosis (pertaining to the cardiovascular disease category), connective tissue disorder, and cell growth and proliferation were persistently upregulated at all temporal studied stages.

**Figure 5:**
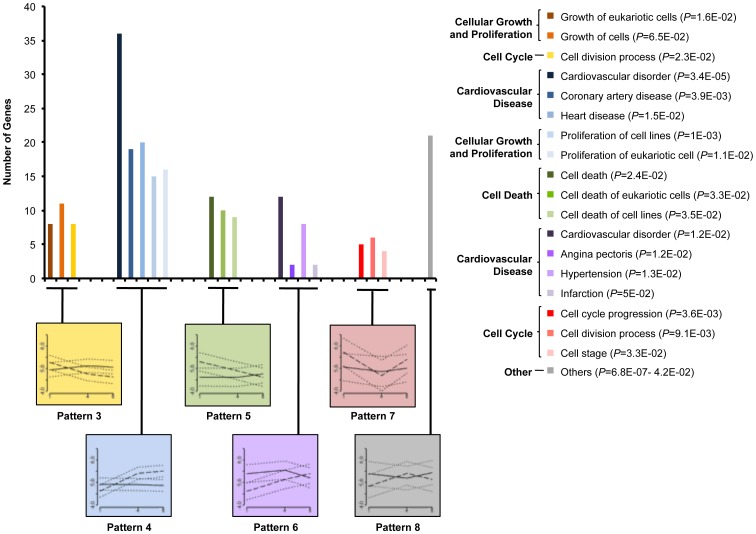
Secondary temporal patterns of differentially expressed genes in infarct core tissue clustered within functional groups. Representative categories, divided in function annotations, are plotted. Related temporal expression patterns of the original probe sets over time are also showed in the corresponding graphs at bottom. For each temporal pattern, mean of log_2_ FC for probe sets with 95% CI were computed. Solid line = remote myocardium, dashed line = infarct core and dotted lines = 95% CI for mean. X axis relates to weeks and Y axis relates to log_2_ FC. *P* value ranges for function annotations are included in the graph legend.

Six additional temporal patterns (patterns 3 to 8) were identified, including a total of 411 regulated probe sets (Fig. 5). Pattern 3 and 4 comprised probe sets that were up- or downregulated, respectively, at week 1 whose expression shifted early, prior to week 4. These included mainly categories of cellular growth and proliferation (45 genes), cell cycle (20 genes), and cardiovascular disease (37 genes). The fifth and sixth patterns of probes included those up- or downregulated, respectively, at week 1 whose expression shifted late by week 4. Here, we mainly found genes involved in cell death (13 genes) and cardiovascular disease (15 genes) categories. Finally, patterns 7 and 8 were defined as genes showing a “V” or “Λ” shape. These are genes upregulated at weeks 1 and 6, and downregulated at week 4 (33 genes, 6 of them associated with cell cycle) or vice versa (42 genes, including 5 genes associated with protein synthesis).

### Validation of microarray results by qRT-PCR

**Figure 6:**
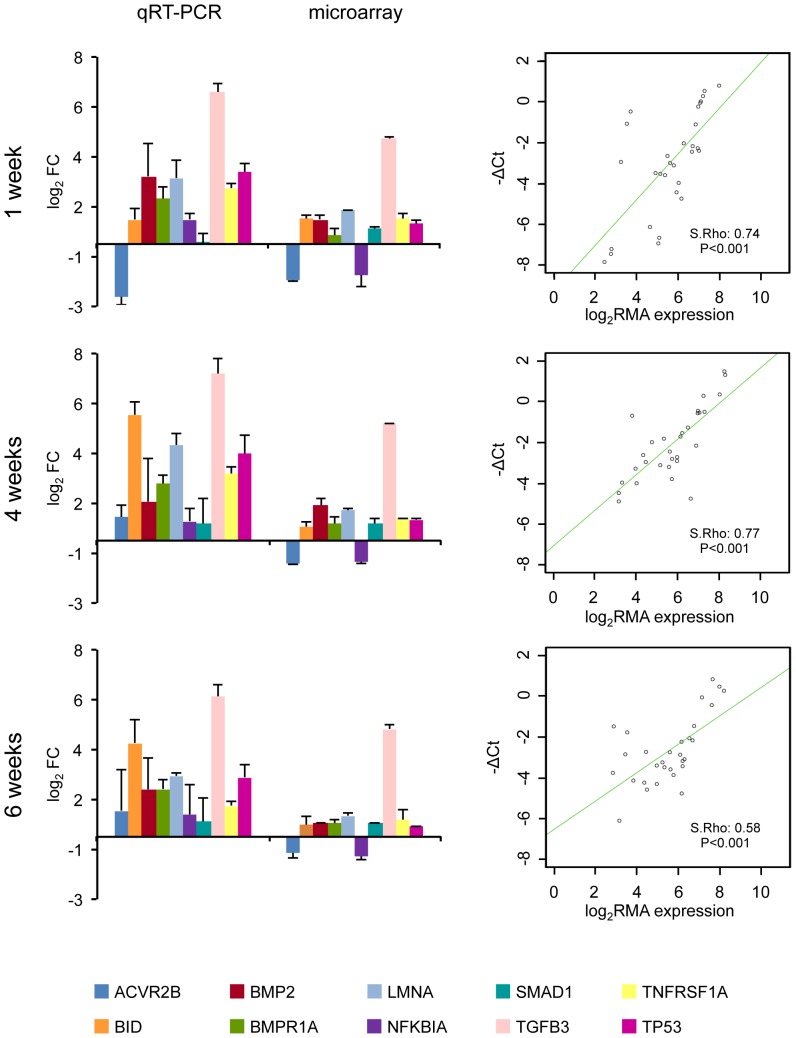
Fold change comparison based on qRT-PCR results and microarray expression data for ten selected genes in the infarct core region. qRT-PCR gene expression signals were normalized to PGK1 housekeeping gene. Data represents mean ± sd of log_2_ FC. Scatter plots of −ΔCt qRT-PCR and microarray log_2_ FC expression with regression lines and Spearman's correlation were showed at 1, 4, and 6 weeks post-MI. FC = fold change.

To validate microarray data, we selected ten genes (ACVR2B, BID, BMP2, BMPR1A, LMNA, NFKBIA, SMAD1, TGFB3, TNFRSF1A, and TP53) from two canonical pathways previously distinguished in the infarct core region and performed qRT-PCR with the same RNA samples obtained for microarray experiments. Significant differences were observed in expression levels of 60% of the validated genes by qRT-PCR between the infarct core and the healthy myocardial regions. Furthermore, Spearman's correlation coefficients of 0.74 (*P*<0.001), 0.77 (*P*<0.001), and 0.58 (*P*<0.001) indicated a moderate-high concordance between microarray results and data obtained by qRT-PCR at 1, 4, and 6 weeks, respectively (Fig. 6).

## Discussion

We evaluated myocardial gene expression alterations due to MI by microarray screening in the swine model. The goal of our study was not to identify all differentially expressed genes between MI and physiological condition, but rather identify sets of genes altered during different stages of LV remodelling, to better understand the underlying molecular basis of the disease. We identified more than 8,000 different probe sets with altered expression in the remodelling myocardium and, by using bioinformatics tools, we recognized functional gene clusters, which may provide much more biological information rather than listing single genes [20]. The cluster analysis showed gene specific expression patterns with related functions both in the infarct core and remote myocardium at 1, 4, and 6 weeks post-MI, revealing molecular footprints associated with different stages of infarct progression. Nevertheless, myocardial remodeling following infarct is an evolving process where many more genes than those found at the different temporal analyzed stages (1, 4, and 6 weeks) could be altered. Results obtained by microarray were also supported by verification of the expression of ten de-regulated genes by qRT-PCR.

Several array-based studies of human HF have distinguished transcripts with altered expression [21]–[27]. Most of the human myocardial samples have been acquired as biopsies with different conservation method, from rejected hearts for transplantation or as explants of diseased myocardium. Furthermore, some studies have shown that cardiomyopathy of different aetiologies exhibits different patterns of gene expression [22], [28]. Contrary, one study comparing the gene expression of ischemic and non-ischemic cardiomyopathy found no differentially expressed genes [29]. Thus, mixed aetiology of these studies together with different ages and sex of patients, have contributed to increase the biological variability of the samples making extremely difficult to determine simply by arrays which gene products are responsive to HF.

Large animal models, with a close physiology to humans, are essential to study the pathophysiological changes associated with ischemic heart disease before clinical translation. Recently, Kuster and colleagues have performed a transcriptional genomics approach 3 weeks after MI induction in swine [30]. They compared non-infarcted remote myocardium after infarct with myocardial samples from sham-surgery to identify candidate transcription factors mediating the genetic reprogramming involved in post-MI LV remodelling [30]. Here, we examined changes in gene expression at three different temporal stages of infarct progression (1, 4, and 6 weeks) in the porcine model including infarct core, non-infarcted remote myocardium and control myocardium from healthy animals. This was based on previously published microarray data, which identified region-specific gene expression in comparisons of ventricular free wall versus interventricular septum [27], [31]–[33]. Work performed by Walther's group have reported that MI in the rat model triggers B_2_-receptor upregulation in the infarcted left ventricle, as well as in the non-infarcted parts of the heart, the right ventricle and in the interventricular septum, demonstrating that these regions also undergo a remodelling after induction of MI [31]. Importantly, our data obtained from microarray analysis in the porcine post-MI model, the animal model with the highest similarity to the human heart in size and physiology [12], support this hypothesis. We showed that MI alters gene expression not only in the infarct core but also in the remote myocardial region.

Among these de-regulated genes in the whole heart, we identified some genes associated with cardiac diseases like cardiac arteriopathy, arrhythmia, and HF. One of them, NPR1, also NPR-A, is one of the three known single membrane-spanning natriuretic peptide receptors in mammals [34]. The murine NPR-A gene has been disrupted by two separate laboratories obtaining null animals with high blood pressure, cardiac hypertrophy, and ventricular fibrosis [35], [36]. Particularly, mice lacking a functional NPR1 gene coding for NPR-A have elevated blood pressures and hearts exhibiting marked hypertrophy with interstitial fibrosis resembling that seen in human hypertensive heart disease [36]. Furthermore, transgenic mice with the NRP1 gene deletion targeted specifically to cardiac tissue exhibited cardiac hypertrophy in the absence of systemic hypertension [37], demonstrating conclusively that NPR1-signaling functions as an intrinsic inhibitor of myocyte growth. Cardiac fibrosis is a classical feature of hypertrophy and is characterized by the expansion of the ECM due to the accumulation of collagen, particularly collagen types I and III [38]. Moreover, TGF-β promotes the proliferation of fibroblasts, stimulates ECM protein production while inhibiting its degradation by induction of antiproteinases or reduction of metalloproteases [38]. It has been demonstrated that TGF-β1 induces collagen I and III mRNA in atrial samples and in isolated cardiac fibroblasts [39], and transgenic mice overexpressing TGF-β1 develop cardiac hypertrophy and interstitial fibrosis [40]. Here, we found NPR1 gene downregulated early 1 week post-MI both at remote myocardium and infarct core regions, and TGF-β1, TGF-β3, and collagen types I and III genes upregulated only in the infarct core tissue at this temporal stage. These findings suggest that NPR1 could trigger hypertrophic pathways in the whole heart and TGF-β signalling may promote interstitial fibrosis via stimulating collagen types I and III gene transcription in the infarcted myocardial region.

Although we observed a marked altered gene expression in the infarct core region, there were 28 de-regulated genes exclusively in the remote myocardium in at least one of the three temporal analyzed stages. Importantly, some of these genes were associated with cardio-related functions, i.e. SRF. Several recent findings from transgenic studies and human disorders indicate the importance of SRF in the myocardium. Heart-specific overexpression of SRF in transgenic mice led to the development of cardiac hypertrophy and cardiomyopathy [41], whereas overexpression of a mutant dominant-negative form of SRF led to a severe dilated cardiomyopathy [42]. Interestingly, here we demonstrated for the first time overexpression of SRF early at 1 week post-MI not only in the infarct core but also in the remote myocardial region.

The nature and extent of gene expression varied with time. When coronary occlusion lasts from 24 hours to several weeks, the injured myocardium responds with increased expression of genes associated with apoptosis and ECM production and decreased expression of energy production-related genes [5], [43]–[45]. Accordingly, here we found 48 de-regulated genes in infarct core region promoting cellular apoptosis, including 22 pro-apoptotic overexpressed genes (e.g. APAF1, BAX, BCL2L11, BID, CASP2, CASP3, CASP6, HTRA2, and TP53) and 22 anti-apoptotic downregulated genes (e.g. BCL2L1, BIRC2, BIRC3, BIRC6, ENDOG, HERAS and XIAP) over time. Moreover, several ECM remodelling-related genes (e. g. collagens, integrins, matrix metalloproteinases, and metalloproteinases inhibitors) showed steady upregulation up to 6 weeks post-MI in the infarct core region. In addition, genes involved in lipid metabolism with prevalence of those involved in catabolism of fatty acids (e.g. enoyl-coenzyme A (CoA) isomerase, dienoyl-CoA reductase, hydroxyacyl-CoA dehydrogenase, ketoacyl-CoA thiolase), were also consistently repressed until 6 weeks post-infarction.

The apoptotic mechanisms are complex and involve different energy-dependent molecular events, including signals which regulate apoptosis via the extrinsic pathway (death receptor pathway), and intracellular inducers regulating the intrinsic apoptotic signalling pathway (mitochondrial pathway) [46]. The extrinsic pathway begins outside the cell through activation of pro-apoptotic receptors on the cell surface, no depending on the cellular stress signals like ischemia, hyperthermia, and toxins as intrinsic pathway does. Normally, in this intracellular pathway pro-apoptotic proteins released from the mitochondria activate caspase proteases and trigger apoptosis. Interestingly, there is an extrinsic-intrinsic pathway overlap and integration due to BID, a pro-apoptotic member of the Bcl-2 family. BID activates caspase-9, an initiator caspase that activates the executing caspases directly responsible for cell death itself [47]. However, in the majority of conditions this overlap is minimal, and the two routes operate independently [48]. According to this, we could expect that most of de-regulated apoptotic genes found in our model after MI belonging to intrinsic pathway. However, BCL2L11, BID, TNFRSF1A and TNFRSF1, genes related to extrinsic pathway, were also de-regulated. A recent study described BAX, a gene activated by BID [49], as a new target of gene therapy to reduce infarct size and improve ventricular function after MI [50]. This idea reinforces our findings which elucidate that upregulation of BID could activate BAX pro-apoptotic function. Further research, both *in vitro* and *in vivo*, is required to assess whether controlling BID apoptotic signaling may contribute to improve cardiac function after MI. In our study, some TRs were altered over time in the infarct core region. We detected TWIST1 overexpressed until 6 weeks post-MI. The Twist family of basic helix-loop-helix (bHLH) transcription factors including TWIST1/2, HAND1/2, Scleraxis, and Paraxis, play a variety of roles in both embryonic development and diseases [51]–[53]. A study performed with transgenic mice overexpressing HAND1 and TWIST1 in cardiomyocytes reported pathological cardiac remodelling leading to HF and sudden death [54]. On the other hand, PPARD is a critical regulator of fatty acid oxidation in cardiac tissue and its lack of expression has been associated with the onset of cardiac failure [55]. Indeed, mice with cardiac-specific deletion of PPARD develop age-dependent cardiac lipotoxicity, cardiac hypertrophy, end-stage dilated cardiomyopathy, and decreased survival [55]. Here we demonstrated persistently downregulation of this TR at all temporal analyzed stages in the infarct core region. These results suggest that TWIST1 could trigger pathological cardiac remodelling after infarction and PPARD may contribute to cardiac hypertrophy development early post-MI.

Gene expression analysis is considered hypothesis generating until validated by another technique. The reliability and quality of microarray results depend on several factors such as sample size, array production, RNA extraction, probe labelling, hybridization conditions, and image analysis. Therefore, differentially expressed genes identified by this method require further validation using another independent technique. qRT-PCR is the most common technique used for microarray data validation, which is a quantitative, rapid, and sensitive method that requires 1,000-fold less RNA compared to conventional assays for gene expression studies [56]–[58]. Here, we confirmed the level of transcript abundance of ten genes using qRT-PCR independent of the microarray platform, and the FC agreed in all cases and 60% were confirmed statistically by a Student's t-test. It is widely known that expression data, i.e. mRNA levels, may not accurately reflect protein levels, since translational control and post-translation processing may occur [59]. In turn, protein expression may not always have a physiological or pathological consequence. However, use of DNA microarrays facilitates systematic exploration of gene expression on a genome-wide scale and should yield wide information about the physiology and pathology of the heart. Furthermore, comparisons of gene expression changes among different models should contribute to a greater understanding of the relationship between genes and disease. Studies focusing on the discovery of specific response pathways, identification of factors promoting the activation of protective genetic factors within cardiomyocytes, and factors downregulating injury-related genes should be a priority in developing new therapeutic strategies to overcome the deleterious consequences of an ischemic event.

## Supporting Information

Figure S1 **Tissue-specific gene expression in MI.** **a** Venn diagrams summarizing differentially altered probe sets in at least one of the three temporal analyzed post-MI stages and **b** those simultaneously altered at 1, 4, and 6 weeks after infarction. IC = infarct core; RM = remote myocardium; n = 3 per group.
(TIF)

Figure S2 **Altered canonical pathways in the infarct core tissue commonly identified at 1, 4, and 6 weeks post-MI (2).** Schematically illustrations of hypoxia signaling in the cardiovascular system, human embryonic stem cell pluripotency, and cardiac hypertrophy signalling pathways at each temporal considered stage. Both upregulated (red filled) and downregulated (green filled) molecules are shown.
(TIF)

Table S1 **qRT-PCR Taqman® Gene Expression assays for porcine genes.**
(DOCX)

Table S2 **Differentially expressed probe sets in the remote myocardium compared with control tissue at 1 week post-MI.**
(XLSX)

Table S3 **Differentially expressed probe sets in the infarct core compared with control tissue at 1 week post-MI.**
(XLSX)

Table S4 **Differentially expressed probe sets in the remote myocardium compared with control tissue at 4 weeks post-MI.**
(XLSX)

Table S5 **Differentially expressed probe sets the in infarct core compared with control tissue at 4 weeks post-MI.**
(XLSX)

Table S6 **Differentially expressed probe sets in the remote myocardium compared with control tissue at 6 weeks post-MI.**
(XLSX)

Table S7 **Differentially expressed probe sets in the infart core compared with control tissue at 6 weeks post-MI.**
(XLSX)

Table S8 **Transcription regulators altered at 1, 4, and 6 weeks after infarction in the infarct core region.** *P* values of overlap predicted by IPA are showed. TR = transcription regulator; FC = fold change. n.a. = non-altered expression; bold green = downregulated over time; bold red = upregulated over time; n.p. = non-predicted.
(DOCX)
