# Supplementary figures and images for: Identification of Temporal and Region-Specific Myocardial Gene Expression Patterns in Response to Infarction in Swine
Source: PLoS One. 2013 Jan 25;8(1):e54785. doi: 10.1371/journal.pone.0054785 (PMC3556027; doi:10.1371/journal.pone.0054785)

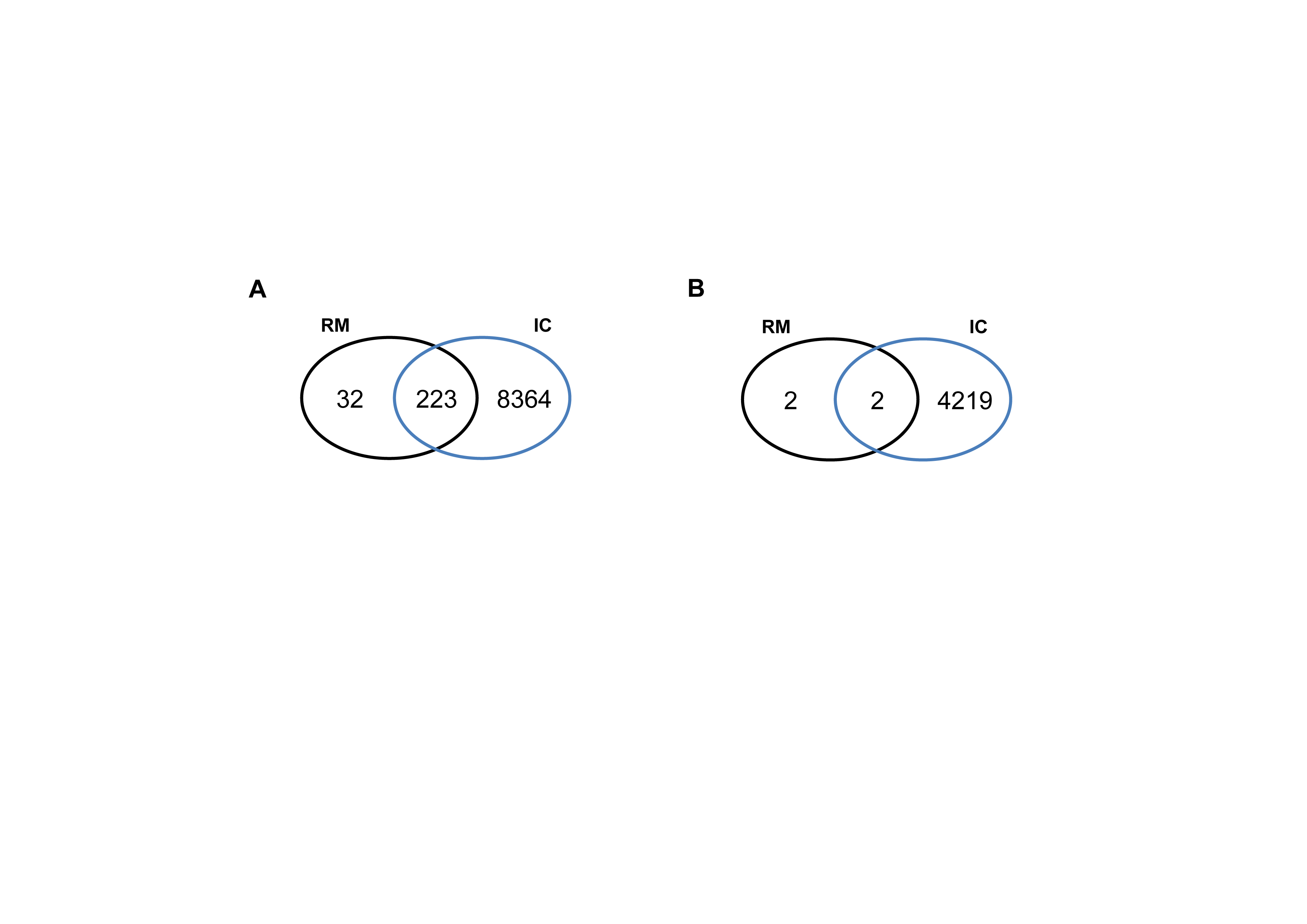

Supplement: Figure S1 — Tissue-specific gene expression in MI. a Venn diagrams summarizing differentially altered probe sets in at least one of the three temporal analyzed post-MI stages and b those simultaneously altered at 1, 4, and 6 weeks after infarction. IC = infarct core; RM = remote myocardium; n = 3 per group. (TIF) [file pone.0054785.s001.tif]

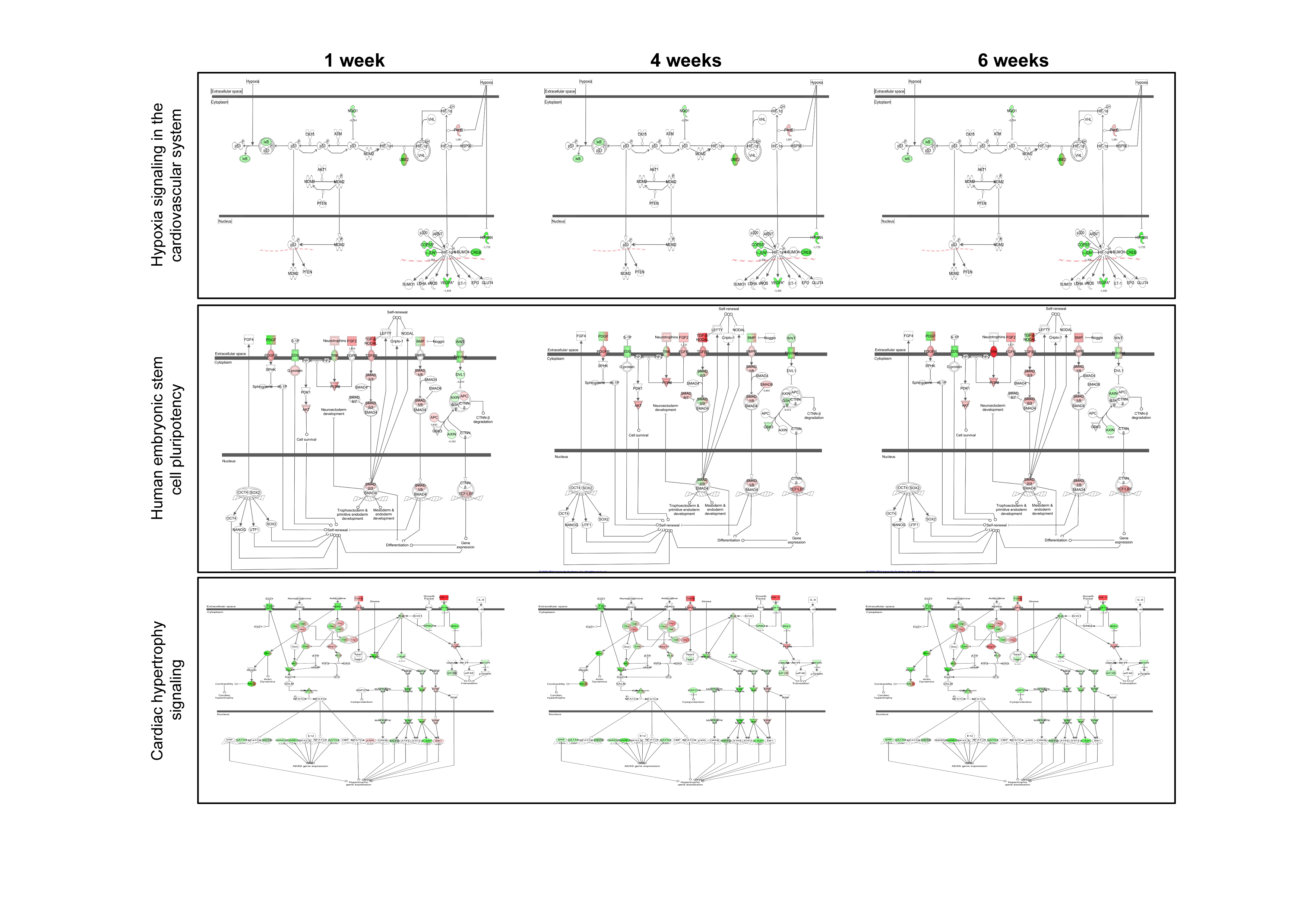

Supplement: Figure S2 — Altered canonical pathways in the infarct core tissue commonly identified at 1, 4, and 6 weeks post-MI (2). Schematically illustrations of hypoxia signaling in the cardiovascular system, human embryonic stem cell pluripotency, and cardiac hypertrophy signalling pathways at each temporal considered stage. Both upregulated (red filled) and downregulated (green filled) molecules are shown. (TIF) [file pone.0054785.s002.tif]
