# Supplementary material for: Identification of Temporal and Region-Specific Myocardial Gene Expression Patterns in Response to Infarction in Swine
Source: PLoS One. 2013 Jan 25;8(1):e54785. doi: 10.1371/journal.pone.0054785 (PMC3556027; doi:10.1371/journal.pone.0054785)
Supplement: Table S1 — qRT-PCR Taqman® Gene Expression assays for porcine genes. (DOCX) [file pone.0054785.s003.docx]

**Supplementary Table S1**. qRT-PCR Taqman^®^ Gene Expression assays for porcine genes.

| **Porcine Gene** | **Taqman assay** |
| --- | --- |
| ACVR2B | Ss03391775_m1 |
| BID | Ss03391974_m1 |
| BMP2 | [Ss03373798_g1](https://products.appliedbiosystems.com/ab/en/US/adirect/ab?cmd=ABAssayDetailDisplay&assayID=Ss03373798_g1&Fs=y&adv_phrase3=EXACT&adv_phrase2=EXACT&adv_phrase1=EXACT&assayType=GE&catID=601267&SearchRequest.Common.SortSpec=RECOMMENDED_ASSAY_FLAG+desc&searchValue=null&searchBy=null&adv_kw_filter3=ALL&srchType=keyword&adv_kw_filter2=ALL&SearchRequest.Common.QueryText=BMP2&kwdropdown=ge&adv_kw_filter1=ALL&species=scrofa&adv_query_text3=&searchType=keyword&adv_query_text2=&adv_query_text1=&adv_boolean3=AND&displayAdvSearchResults=null&adv_boolean2=AND&adv_boolean1=AND&chkBatchQueryText=false&kwfilter=ALL&SearchRequest.Common.PageNumber=1&isSL=null&msgType=ABGEKeywordResults) |
| BMPR1A | Ss04248558_m1 |
| SMAD1 | Ss03391094_m1 |
| TGFB3 | Ss03394350_m1 |
| LMNA | Ss03389729_m1 |
| NFKBIA | Ss03391764_m1 |
| TNFRSF1A | Ss03391125_m1 |
| TP53 | Ss04248636_m1 |
| GUSB | Ss03387751_u1 |
| GAPDH | Ss03375435_u1 |
| PGK1 | Ss03389144_m1 |
