# Supplementary material for: Identification of Temporal and Region-Specific Myocardial Gene Expression Patterns in Response to Infarction in Swine
Source: PLoS One. 2013 Jan 25;8(1):e54785. doi: 10.1371/journal.pone.0054785 (PMC3556027; doi:10.1371/journal.pone.0054785)
Supplement: Table S8 — Transcription regulators altered at 1, 4, and 6 weeks after infarction in the infarct core region. P values of overlap predicted by IPA are showed. TR = transcription regulator; FC = fold change. n.a. = non-altered expression; bold green = downregulated over time; bold red = upregulated over time; n.p. = non-predicted. (DOCX) [file pone.0054785.s010.docx]

**Supplementary Table S8**. Transcription regulators altered at 1, 4, and 6 weeks after infarction in the infarct core region. *P* values of overlap predicted by IPA are showed. TR=transcription regulator; FC=fold change.

| **TR** | **1 week** | | **4 week** | | **6 week** | |
| --- | --- | --- | --- | --- | --- | --- |
|  | **log_2_FC** | ***P* value of overlap** | **log_2_FC** | ***P* value of overlap** | **log_2_FC** | ***P* value of overlap** |
| CEBPB | n.a. | - | -0.99 | 6.9E-03 | n.a | - |
| **EPAS1** | **-1.16** | **1.2E-02** | **-1.04** | **4.8E-02** | **-1.37** | **1.9E-02** |
| EZH2 | n.a. | - | n.a. | - | 1.55 | 1.3E-01 |
| **HEY1** | **-0.72** | **6.9E-03** | **-0.93** | **4.4E-02** | **-1.06** | **n.p.** |
| **HEY2** | **-2.37** | **3.7E-02** | **-1.89** | **3.6E-02** | **-1.88** | **2.4E-02** |
| HIF1A | 0.80 | 1.9E-02 | n.a. | - | n.a. | - |
| HMGA1 | n.a. | - | -0.91 | 7.1E-03 | -0.50 | n.p. |
| IRF3 | 0.36 | 2.7E-02 | n.a. | - | n.a. | - |
| **JARID1B** | **0.85** | **4.8E-02** | **0.82** | **1.1E-02** | **0.87** | **3.4E-02** |
| **JUN** | **-1.83** | **3.4E-02** | **-1.47** | **3.8E-02** | **-1.37** | **n.p.** |
| KLF11 | -1.97 | 3.7E-02 | n.a. | - | n.a. | - |
| **MBD2** | **1.30** | **4.4E-01** | **0.91** | **n.p.** | **0.61** | **n.p.** |
| MLL2 | n.a. | - | -0.39 | 5.7E-03 | n.a. | - |
| **MYOCD** | **-1.88** | **4.8E-02** | **-1.56** | **1.7E-02** | **-1.24** | **n.p.** |
| MZF1 | n.a. | n.a. | -0.43 | 1.7E-02 | n.a. | n.a. |
| **NOTCH3** | **0.82** | **8E-02** | **1.13** | **9.8E-02** | **0.76** | **5.2E-02** |
| **PPARD** | **-0.58** | **2.4E-01** | **-0.63** | **2.4E-01** | **-0.36** | **3.1E-01** |
| **PPARGC1A** | **-3.13** | **1E00** | **-3.10** | **4.1E-01** | **-1.42** | **5.6E-01** |
| RB1 | n.a. | - | -0.81 | 3.7E-01 | -0.52 | 4.7E-01 |
| RELA | 0.31 | n.p. | 0.40 | 1.7E-02 | n.a. | - |
| **SATB1** | **-1.29** | **n.p.** | **-2.19** | **n.p.** | **-1.38** | **3.8E-02** |
| **SMAD1** | **0.60** | **1E-01** | **0.66** | **9.8E-02** | **0.55** | **6.1E-02** |
| SMAD3 | n.a. | - | -0.59 | 3.8E-02 | n.a. | - |
| SMARCA4 | 1.10 | 3.5E-02 | 0.37 | 3.7E-01 | n.a. | - |
| STAT6 | 0.76 | n.p. | 0.72 | 2.8E-02 | n.a. | - |
| TP53 | 0.82 | 2.7E-03 | 0.84 | 1.4E-05 | n.a. | - |
| **TWIST1** | **1.47** | **1.1E-01** | **2.00** | **1E-01** | **1.64** | **4.7E-02** |

n.a.=non-altered expression; bold green=downregulated over time; bold red=upregulated over time; n.p.=non-predicted.
